# Draft Genome Sequence of Strain CBD-635, a Methicillin-Resistant *Staphylococcus aureus* USA100 Isolate

**DOI:** 10.1128/genomeA.00491-13

**Published:** 2013-07-11

**Authors:** Ronan K. Carroll, Whittney N. Burda, Jill C. Roberts, Kealy K. Peak, Andrew C. Cannons, Lindsey N. Shaw

**Affiliations:** Department of Cell Biology Microbiology and Molecular Biology, University of South Florida, Tampa, Florida, USAa; Department of Environmental and Occupational Health, University of South Florida, Tampa, Florida, USAb; Florida Department of Health, Tampa, Florida, USAc; Center for Biological Defense, Department of Global Health, University of South Florida, Tampa, Florida, USAd

## Abstract

We present the draft genome sequence of methicillin-resistant *Staphylococcus aureus* strain CBD-635, from the USA100 lineage. This is a sepsis isolate obtained from Tampa General Hospital. This strain is *spa* type t003 and multilocus sequence typing (MLST) type ST5, and it has been used by our group in the study of novel antimicrobial chemotherapeutics.

## GENOME ANNOUNCEMENT

*Staphylococcus aureus* is a highly virulent, Gram-positive bacterial pathogen, capable of causing a variety of ailments throughout the human body. The emergence of methicillin-resistant *S. aureus* (MRSA) strains that are resistant to last-resort antibiotics demonstrates the urgent need for new treatments and therapies to prevent and treat *S. aureus* infections. Previously, we have performed studies to identify novel antimicrobial compounds that could be used to treat strains of MRSA that are resistant to multiple classes of antibiotics (1–5). In these studies, we employed an extensively drug-resistant strain of MRSA (strain CBD-635) isolated from a sepsis patient at Tampa General Hospital. Pulsed-field gel electrophoresis (PFGE) analysis demonstrated that CBD-635 is from the USA100 lineage and that the isolate is resistant to a variety of antimicrobial compounds, including methicillin, centhromycin, azithromycin, erythromycin, clindamycin, ampicillin, chloramphenicol, gentamicin, tetracycline, and ciprofloxacin, and demonstrates intermediary resistance to daptomycin, linezolid, and vancomycin. The use of strain CBD-635 in our drug discovery studies has facilitated the identification of promising new compounds to treat infections with multiply resistant bacteria (1–5). To gain insight into the mechanism of action of potential new antimicrobial agents, we have generated CBD-635 mutants that exhibit resistance to the new compounds (5). Whole-genome sequencing will be employed to identify the mutation(s) that gives rise to resistance and therefore help elucidate the mechanism of action. To generate a reference genome for these studies, we have sequenced the genome of strain CBD-635 using Illumina next-generation DNA sequencing.

CBD-635 was grown in tryptic soy broth (TSB) overnight and genomic DNA extracted using standard methods (cells were lysed by bead beating and the genomic DNA was extracted using phenol/chloroform). Genome sequencing was performed using an Illumina HiScan SQ, which generated 8 million paired-end reads of 100 bp with an insert size of 300 bp. Of the reads generated, 25% (2 million) were randomly selected and used in a *de novo* genome assembly using the CLC Genomics Workbench software package (CLC, Denmark). This assembly generated 86 contigs with a size of >200 bp. The 86 contigs were deposited in GenBank and annotated using the NCBI Prokaryotic Genomes Automatic Annotation Pipeline (PGAAP) (http://www.ncbi.nlm.nih.gov/genome/annotation_prok/). The cumulative size of the genome is approximately 2.82 Mb, encoding 2,686 open reading frames (ORFs), comparable with similar *S. aureus* strains. Using the annotated sequence data, we performed *spa* typing and multilocus sequence typing (MLST) analysis for CBD-635. Similar to other sequenced USA100 isolates, CBD-635 is *spa* type t003 and MLST type ST5.

### Nucleotide sequence accession number.

The sequence data for this genome have been deposited in GenBank under the accession number ASHS00000000.
